# Fracture of the Orbit from a Lead Pencil

**Published:** 1848-08

**Authors:** 


### PATHOLOGICAL SOCIETY OF LONDON.

Mr. Hughes Hewett exhibited a specimen of *Fracture of the orbit from a lead pencil.*—A child, two years old, fell on crossing the room with a lead pencil in her hand. The pencil penetrated the right orbit, having pierced the eyelid just below the supra-orbital ridge. The eye did not appear injured in any way, and there were no symptoms of concussion. Considerable force was required to extract the pencil, which had penetrated to the depth of about two inches. Fomentations were applied to the wound, which closed in twenty-four hours. The child ate its dinner much as usual soon after the accident, and slept well during the night. Next morning it was drowsy; it was seen for the first time by the medical attendant of the family, who applied a leech to the temple, and ordered calom. gr. ss. 6tis. horis. On the middle of the next day (the 3rd), she had a convulsion, twitching of the muscles of the face, legs, arms, &c., after which she rapidly lost consciousness, sight, and hearing. Mr. Hewett saw her for the first and only time on the fourth day; she was then lying on her back; her countenance perfectly placid; the right eye ecchymosed and closed, and a muco-purulent secretion was issuing from between the lids; there were frequent subsultus of the fingers and hands, sighing, and a peculiar clicking sound produced by moving the tongue between the lips; she could not be roused at all. (The calomel was stopped, having caused considerable diarrhœa). The pupil of the left eye was active, but acted quite independently of the stimulus of light: it dilated when the eyelid was suddenly raised. The left cheek became flushed, and the child was generally feverish towards night. Next day (fifth), she was in about the same state, excepting uncontrollable diarrhœa, and flushing of both cheeks in the evening. On the sixth day the countenance was cadaveric; she started frequently, picked the bed-clothes, and screamed occasionally when moved. In the evening trismus came on, with cough, difficult deglutition and respiration, almost constant convulsive movements, and drawing of the thumbs across the palms of the hands. For some time before death the right eyelids had been separated, and the eye was seen uninjured, and moving symmetrically with the other. She died early on the morning of the seventh day.

The head was examined. The arachnoid was healthy, but beneath it there was most extensive effusion of greenish yellow lymph, mixed with serum, filling up all the sulci, and in some places concealing the surface of the hemispheres; the arachnoid could be peeled from off it; it was found everywhere, except at the base. The substance of the brain presented nothing remarkable; there was no fluid in the ventricles. On raising the right anterior lobe from the roof of the orbit, its inferior surface was found to be in a softened, broken down, and pulpy state, and upon careful examination a tolerably distinct cavity was discovered, which had probably formed the cyst of an abscess. There was a fracture of the roof of the orbit, and a fissure running nearly from before backwards. The two pieces of bone in the dry preparation were held completely *in situ* by dura mater and the periosteum of the orbit. On pressing the globe of the eye, matter flowed from the orbit through the fissure into the cavity of the cranium. The fissure was a little more than a line in width, and would therefore have scarcely admitted the blunt conical point of a lead pencil. It is probable, however, that its edges were separated to a much greater extent at the time of the accident, and were brought nearly in apposition again by the forcible extraction of the pencil.

